# Prevalence, antibiotic susceptibility, and genomic analysis of *Vibrio alginolyticus* isolated from seafood and freshwater products in China

**DOI:** 10.3389/fmicb.2024.1381457

**Published:** 2024-07-10

**Authors:** Yanan Sun, Yanfei Yan, Shaofei Yan, Fengqin Li, Ying Li, Lin Yan, Dajin Yang, Zixin Peng, Baowei Yang, Jiali Sun, Jin Xu, Yinping Dong, Yao Bai

**Affiliations:** ^1^NHC Key Laboratory of Food Safety Risk Assessment, China National Centre for Food Safety Risk Assessment, Beijing, China; ^2^School of Public Health, Shandong University, Jinan, China; ^3^College of Food Science and Engineering, Northwest A&F University, Xianyang, China

**Keywords:** *Vibrio alginolyticus*, antibiotic resistance, virulence genes, aquatic products, MLST

## Abstract

**Introduction:**

This study characterized *Vibrio alginolyticus* isolated from seafood and freshwater products in China (2020).

**Methods and Results:**

In total, 122 (95.31%) *V. alginolyticus* isolates were resistant to at least 1 antibiotic category, and 2 (1.56%) isolates were resistant to at least 3 antibiotic categories and belong to multi-drug resistance (MDR) isolates. A high prevalence rate was observed to be *blaCARB* (98.04%) encoding beta-lactam resistance, followed by *tet* (97.06%) encoding tetracycline resistance and *fos* (4.90%) encoding resistance to fosfomycin. Among the 57 *V. alginolyticus* isolates, the commonest virulence genes were type III secretion system translocated gene *vopD*, *vopB*, and *vcrH* (54.4%, 31/57), type III secretion system regulated gene *tyeA* (54.39%), followed by *vscI* and *vscF* (50.88%) encoded type III secretion system inner rod protein and needle protein, respectively. Multilocus sequence typing (MLST) showed considerable genetic diversity, with 34 distinct sequence types (STs) identified among 55 isolates. ST421 (*n* = 5), ST166 (*n* = 4), ST523 (*n* = 3), ST516 (*n* = 3), and ST507 (*n* = 3) were dominant STs among 55 *V. alginolyticus* isolates.

**Discussion:**

These findings highlight the widespread occurrence of *V. alginolyticus* in both freshwater and seafood products, underscoring the critical need for vigilant monitoring of these bacteria. Such measures are essential for ensuring effective food safety management and safeguarding public health.

## 1 Introduction

As an opportunistic pathogen, *Vibrio alginolyticus* is a Gram-negative bacterium that is found in marine animals and humans. Fish (Rigos and Katharios, 2010), crustaceans (Liu et al., 2004), and mollusks (Luna-González et al., 2002) are commonly associated with *V. alginolyticus*. Meanwhile, *V. alginolyticus* is a notable human enteropathogen associated with seafood-borne mortality and illness worldwide (Mizan et al., 2017).

With the gradual improvement of people’s living standards and the fast-growing industry for food production, aquaculture supplies over 50% of aquatic protein sources to fulfill human needs (Little et al., 2016; Norman et al., 2019). Approximately 6–8% of the total food-borne diseases are associated with fish, which is greater than the incidence of food illness cases from chicken and beef (Sheng and Wang, 2021). Moreover, pathogenic infections by bacteria cause serious disease outbreaks leading to great economic loss, which hampers sustainable development of the aquaculture industry globally (Ghittino et al., 2003; Stentiford et al., 2012; Yang et al., 2022). In European marine aquaculture, *Vibrio alginolyticus* is a common inhabitant of fish and bivalve hatcheries, and it has been linked to larval mortality when poor water quality favors its growth (Pantelis et al., 2017). The pathogen is particularly relevant to the Chinese aquaculture industry where it has been associated with severe economic damage (Yang et al., 2022).

It has been estimated that 1.9 million people die each year as a result of food- and water-borne illnesses in developing countries (Schlundt et al., 2004). Approximately one-third of the population suffers from microbiological food-borne diseases each year in developed countries (Andargie et al., 2008). *V. alginolyticus* can be transmitted to humans through raw or undercooked contaminated seafood or direct contact of open wounds or broken skin with contaminated salt or brackish water (Klontz et al., 1988; Dechet et al., 2008). The infection may cause severe soft tissue infections, sepsis, and other extraintestinal infections (Altekruse et al., 2000; Dechet et al., 2008; Scallan et al., 2011; Newton et al., 2012; Pickering et al., 2013).

Antibiotics are commonly used as the primary treatment against *Vibrio alginolyticus* infections in marine aquaculture (Cao et al., 2018). However, the extensive and sometimes inappropriate use of antibiotics can lead to the development of antibiotic resistance and residual antibiotics in the environment. The isolation of *V. alginolyticus* strains with multiple antibiotic resistance has been reported from several recent outbreaks (Mechri et al., 2015; Rameshkumar et al., 2017; Mohamad et al., 2019b).

Whole-genome sequencing (WGS) provides comprehensive genetic information and becomes more accessible and useful for serotyping (Yang et al., 2021). Many studies on genetic characteristics and drug resistance among food-borne pathogens in seafood and freshwater products have been performed worldwide (Campos et al., 2013; Odeyemi and Sani, 2016). Nevertheless, there is nearly no WGS information on *V. alginolyticus* isolates from seafood and freshwater products in China.

The prevalence of antibiotic-resistant microbes (ARMs) has become a worldwide issue in seafood, and many isolates from seafood have shown a higher degree of resistance against a wide range of antibiotics (Nguyen et al., 2014). This study aimed to present the genomic epidemiology and antimicrobial susceptibility of *V. alginolyticus* isolates from 23 types of fish products collected from four different retail outlets (supermarkets, wet markets, restaurants, and online shops) in Shanghai and nine provinces (Fujian, Shandong, Zhejiang, Sichuan, Guangxi, Jiangsu, Liaoning, Guangdong, and Heilongjiang) in China, focusing on microbiological contamination.

## 2 Materials and methods

### 2.1 Sample collection and *V. alginolyticus* isolation

In 2020, a total of 128 *Vibrio alginolyticus* isolates were collected across China. The samples included seafood (*n* = 75), freshwater products (*n* = 51), and other samples (*n* = 2). The samples were collected from Shanghai (*n* = 1) and nine provinces [Fujian Province (*n* = 14), Shandong Province (*n* = 19), Zhejiang Province (*n* = 4), Sichuan Province (*n* = 6), Guangxi Province (*n* = 10), Jiangsu Province (*n* = 25), Liaoning Province (*n* = 22), Guangdong Province (*n* = 10), and Heilongjiang Province (*n* = 17)]. The 129 samples were collected from 4 sampling sites [supermarkets (*n* = 33), wet markets (*n* = 63), restaurants (*n* = 24), online shops (*n* = 6), and unknown (*n* = 2)]. The seafood samples were classified into 12 kinds of fish and products, including salmon (*Salmo salar*) (*n* = 44), Spanish mackerel (*Scomberomorus niphonius*) (*n* = 7), sashimi platter (*n* = 5), corvina (*Larimichthys crocea*) (*n* = 5), cod (*Dissostichus eleginoides*) (*n* = 4), sea bass (*Lateolabrax japonicus*) (*n* = 3), tilapiine (*Oreochromis niloticus*) (*n* = 2), capelin (*Mallotus villosus*) (*n* = 1), turbot (*Scophthalmus maximus*) (*n* = 1), flounder (*Paralichthys olivaceus*) (*n* = 1), tuna (*Thunnus thynnus*) (*n* = 1), and sole fish (*Solea solea*) (*n* = 1). The freshwater products included grass carp (*Ctenopharyngodon idella*) (*n* = 11), carp (*Cyprinus carpio*) (*n* = 10), crucian (*Carassius carassius*) (*n* = 6), black carp (*Mylopharyngodon piceus*) (*n* = 6), perch (*Siniperca chuatsi*) (*n* = 6), loach (*Misgurnus anguillicaudatus*) (*n* = 2), *tilapia mossambica* (*Oreochromis mossambicus*) (*n* = 2), barbel chub (*Spinibarbus sinensis*) (*n* = 2), finless eel (*Monopterus albus*) (*n* = 1), white bream (*Pampus argenteus*) (*n* = 1), Mandarin fish (*Siniperca chuatsi*) (*n* = 1), blue swordfish (*Makaira nigricans*) (*n* = 1), dogfish (*Squalus acanthias*) (*n* = 1), and croaker (*Larimichthys crocea*) (*n* = 1).

*Vibrio alginolyticus* was isolated from the samples of seafood and freshwater products following the protocols of the National Food Safety Standard (GB4789.7-2013) of China. In brief, 25 g of samples were placed in a sterile plastic bag containing 225 mL of buffered peptone water (BPW) and shaken for 2 min. The rinse was placed in an incubator at 36 ± 1°C for 12 h. Subsequently, the 1 mL of rinse was transferred into a tube with 9 mL of BPW for dilution. The dilution was incubated at 36 ± 1°C for 12 h. A loopful of dilution culture was streaked onto thiosulfate–citrate–bile salts–sucrose agar and then incubated at 36 ± 1°C for 24 h. Three colonies with a typical *V. alginolyticus* phenotype were picked from each plate and purified on fresh 3% NaCl tryptic soy agar. After incubation for 24 h at 36 ± 1°C, three typical colonies were selected at random and used for API 20E (bioMérieux, Marcy-lÉtoile, France) identification.

### 2.2 Antimicrobial susceptibility test

The antibiotic susceptibility of *V. alginolyticus* isolates was tested using the broth microdilution method, according to the guidelines issued by the Clinical and Laboratory Standard Institute (CLSI, 2020). Antibiotics were selected and used for antimicrobial susceptibility tests considering the antibiotics commonly used in clinical treatment. A total of 12 antibiotics, each representing a different category, were employed: ampicillin, amoxicillin/clavulanic acid, cefazolin, cefotaxime, ceftazidime, imipenem, gentamicin, tetracycline, ciprofloxacin, levofloxacin, trimethoprim/sulfamethoxazole, and chloramphenicol. This range of antibiotics allowed for a comprehensive evaluation of the resistance profiles of the *V. alginolyticus* isolates across various antibiotic classes.

### 2.3 Whole-genome sequencing

In total, 102 *V. alginolyticus* isolates were selected for WGS. Genomic DNA was extracted using a bacterial Genomic DNA Extraction Kit (TIANGEN Biotech Co., Ltd., Beijing, China) according to the manufacturer’s instructions. Illumina sequencing was conducted by Majorbio Bio-pharm Technology Co. Ltd. (Shanghai, China) using the Illumina HiSeq 2500 platform (Illumina, Santiago, CA, United States). The genome sequences were analyzed using the Centre for Genomic Epidemiology (CGE) website,^1^ and multi-locus sequence typing (MLST) was conducted using the MLST database on the CGE website as well.

### 2.4 Statistical analysis

Statistical analysis of the data was performed using GraphPad Prism software (GraphPad Prism 7.00). The *t*-test was used to test significant differences in the number of positive samples collected from different provinces. The chi-square test was used to evaluate differences in the presence of *V. alginolyticus* depending on food type, marketplace, and provinces. The distribution of *V. alginolyticus* was visualized using a Sankey plot. Differences in the total number of antibiotic resistance genes (ARGs) were assessed using the Kruskal–Wallis test. Spearman’s rank correlation was conducted in the total number of ARGs, provinces, and virulence genes. The differences between variables were considered statistically significant when *P*<0.05. The bacterial isolate originating from Shanghai constitutes an instance, thereby lacking adequate representativeness and precluding any statistical significance.

## 3 Results

### 3.1 Antimicrobial susceptibility

**Figure 1:**
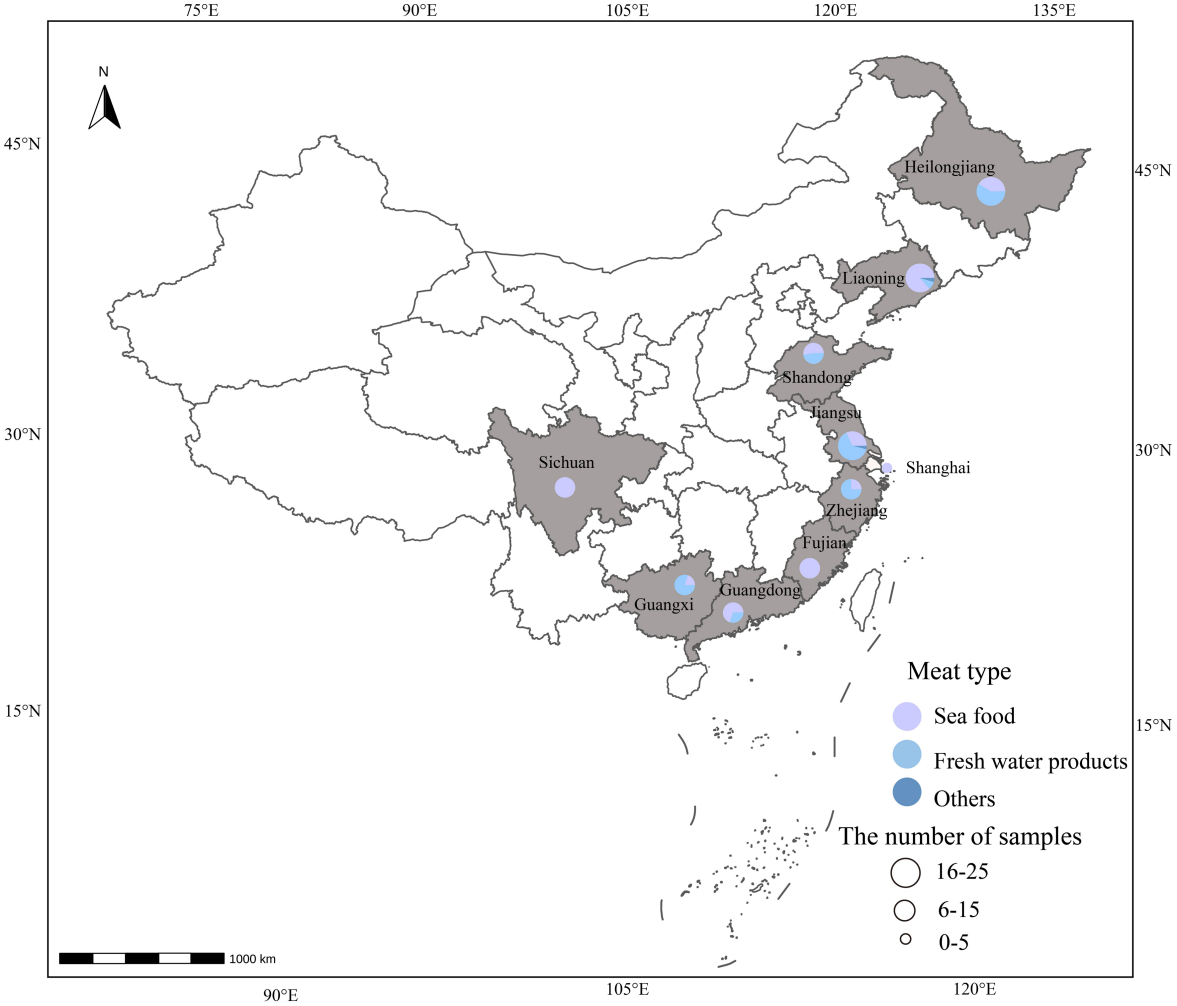
Map of 128 showing 10 sampling provinces of China, 2020. The size of the circle represents a number of samples, and the color represents the sample types.

**Figure 2:**
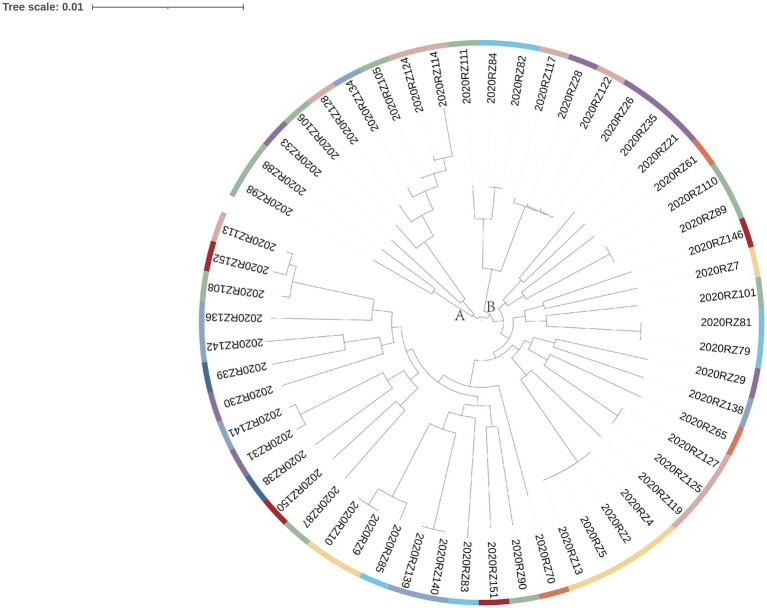
ML tree based on core SNPs. Different colors represent different provinces.

In total, 128 fresh samples were positive for *V. alginolyticus* (Figure 1). To further verify the genetic correlation between *V. alginolyticus* isolates, genomes of 57 *V. alginolyticus* were selected for genetic relatedness analysis (Figure 2). The phylogenetic tree presented in Figure 2 illustrates the genetic diversity among the 57 selected samples. Different colored branches represent different provinces, highlighting the genetic differentiation between various groups. Samples with closer genetic relationships are clustered together, indicating their genetic similarity.

The tree can be primarily divided into two major clusters: A and B. Cluster A comprises samples from Jiangsu Province (four samples), Liaoning Province (three samples), Guangdong Province (one sample), and Shandong Province (one sample). Among the samples in Cluster A, only samples 2020RZ105 and 2020RZ124 are freshwater products, while the others are seafood products. Cluster B can be further subdivided into three categories. Among them, samples 2020RZ122 and 2020RZ26 are the most similar ones, despite originating from seafood from Liaoning Province and freshwater products from Shandong Province, respectively. This suggests that seafood from different provinces might be subjected to contamination from the same source.

**Figure 3:**
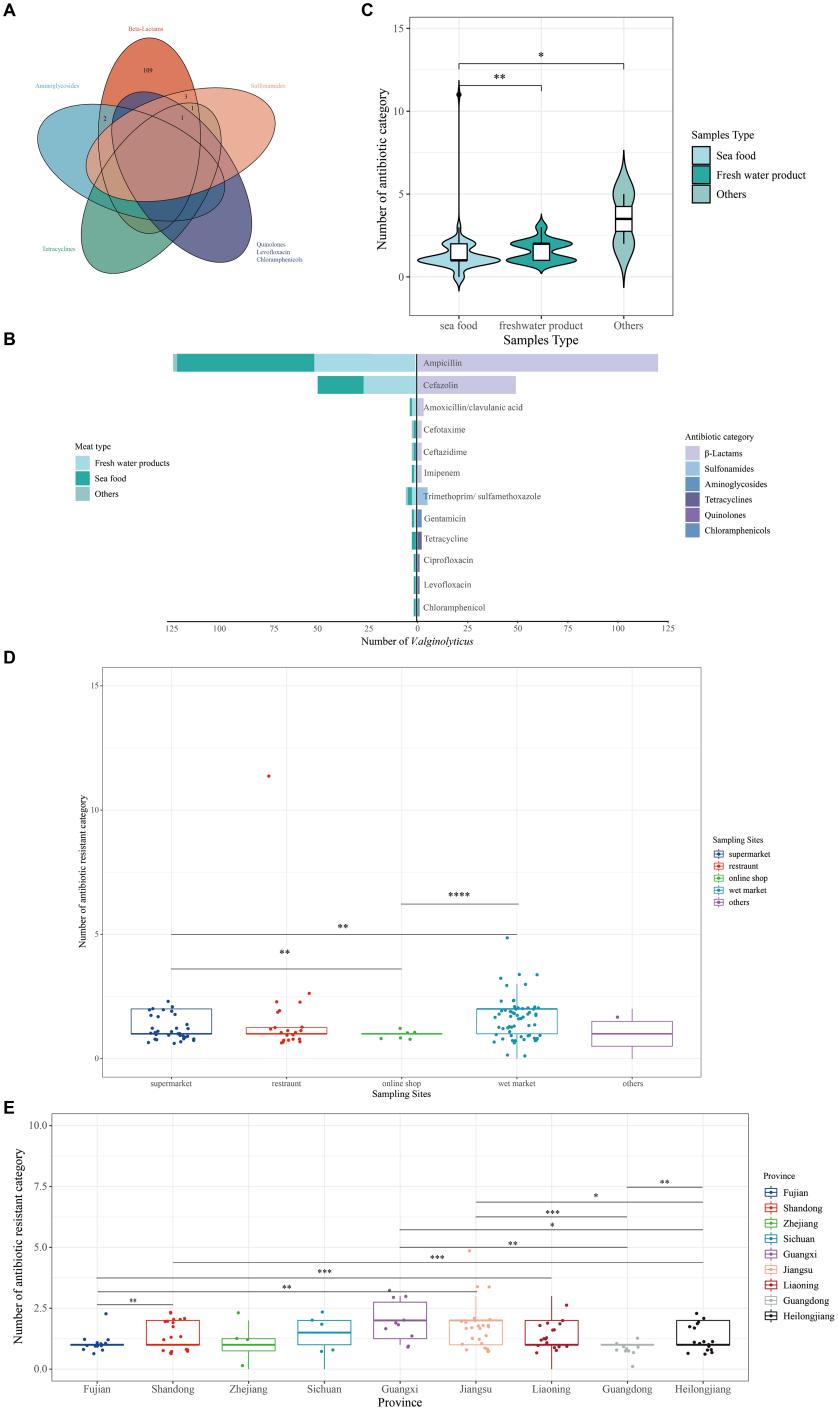
Number of antibiotic-resistant categories of 128 *V. alginolyticus*. **(A)** Venn showing multi-resistant *V. alginolyticus*. **(B)** The category of resistant antibiotics. **(C)** Number of antibiotic-resistant categories among different provinces. **(D)** Antibiotic-resistant categories in different sampling sites. **(E)** Antibiotic-resistant categories in different provinces. *p*-value performed by *.

Of these, 122 (95.31%) *V. alginolyticus* isolates were resistant to at least one category of antibiotic and 2 (1.56%) isolates were resistant to at least three antibiotic categories and belong to MDR isolates (Figure 3A). Resistance to ampicillin (93.75%) was most frequently detected. For the remaining antibiotics, resistance to cefazolin accounted for a large proportion (38.28%), followed by trimethoprim/sulfamethoxazole (3.91%), amoxicillin/clavulanic acid (2.34%), cefotaxime (1.56%), ceftazidime (1.56%), imipenem (1.56%), gentamicin (1.56%), tetracycline (1.56%), ciprofloxacin (0.78%), levofloxacin (0.78%), and chloramphenicol (0.78%) (Figure 3B).

According to meat type, the number of resistant antibiotic category of isolates from seafood was significantly different from freshwater products and two unknown samples (Figure 3C). According to sampling sites, the number of resistant antibiotic category of isolates from the wet market was significantly different from online shop and supermarket (Figure 3D). In terms of the province, the number of antibiotic-resistant isolates from Fujian province was significantly different from Liaoning Province, with Shandong Province significantly different from Heilongjiang Province, and Jiangsu Province significantly different from Guangdong Province (Figure 3E); one sample collected from Shanghai Province was not further analyzed here.

### 3.2 Presence of ARGs and virulence genes in *V. alginolyticus* isolates

**Figure 4:**
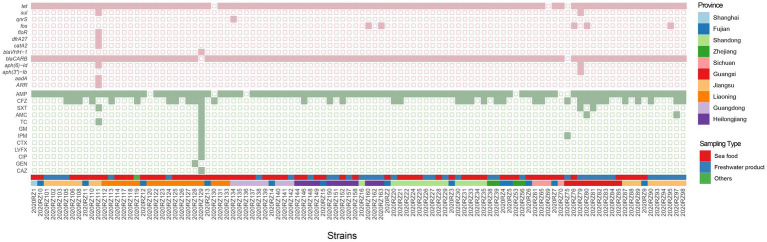
Combined heatmap presenting antibiotic-resistant genes and antibiotic susceptibility of 102 sequenced *V. alginolyticus*. The colors of the squares heatmap represent the detection of antibiotic-resistant genes. Pink squares, positive; light yellow squares, negative. The colors of the circular heatmap represent the detection of antibiotic susceptibility. The size of the circular represents the number of resistant antibiotics. Green, resistant.

In total, 13 antibiotic resistance genes (ARGs) encoding resistance to 10 antimicrobial categories were detected across 102 *Vibrio alginolyticus* isolates (Figure 4). The most common ARG types were found to be involved in resistance to aminoglycosides (*n* = 3) and β-lactams (*n* = 2). For the ARG subtype, a high prevalence rate was observed to be *blaCARB* (98.04%) encoding beta-lactam resistance, followed by *tet* (97.06%) encoding tetracycline resistance and *fos* (4.90%) encoding resistance to fosfomycin. Detection rates of remaining ARGs were all <50%. For instance, the presence of *ARR*, *aadA*, *aph(3″)-Ib*, *aph(6)-Id*, *blaVHH-1*, *catA2*, *dfrA27*, *floR*, *qnrS*, and *sul* was only found in seafood products.

**Figure 5:**
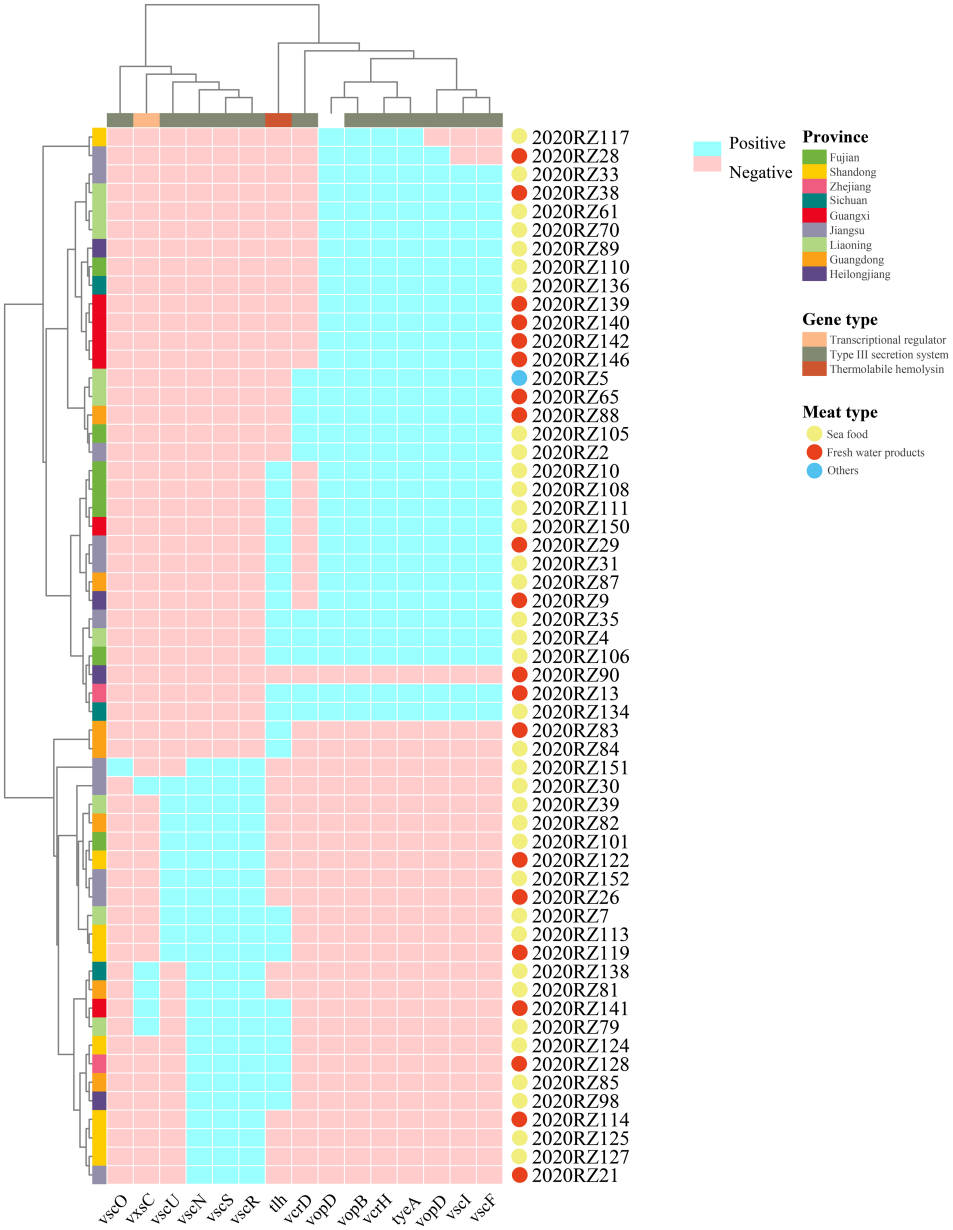
Heatmap and prevalence of virulence genes in different *V. alginolyticus*. Azure in the heatmap represents positive virulence genes. The color of the circle represents meat types.

Furthermore, 15 virulence genes were identified in the 57 *V. alginolyticus* isolates (Figure 5). Among the 57 *V. alginolyticus* isolates, the commonest virulence genes were type III secretion system translocated gene (*vopD*, *vopB*, and *vcrH*), type III secretion system regulated gene *tyeA* (54.39%), followed by *vscI* and *vscF* (50.88%) encoding type III secretion system inner rod and needle proteins, respectively. Detection rates of remaining virulence genes were all <50%. In the type III secretion system class, *vscO* encoding central stalk protein was only present in 2020RZ151. Interestingly, *vscS* and *vscR*, two genes encoding type III secretion system C-ring protein, appeared in pairs.

### 3.3 Phylogenetic analysis

**Figure 6:**
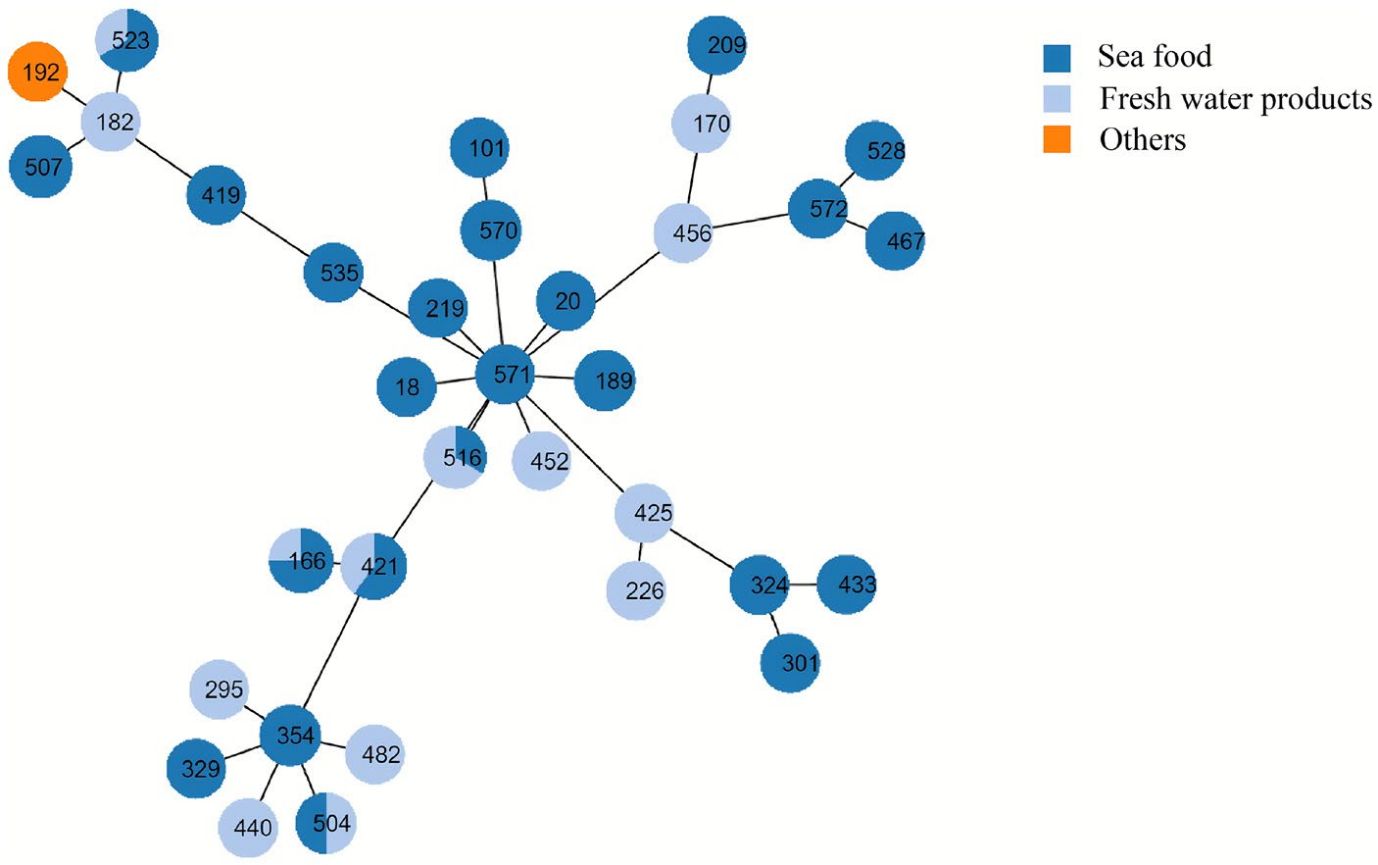
Minimum spanning tree of *V. alginolyticus* isolates by multi-locus sequence typing. Each node represents one ST. The size of the node is related to the number of isolates. Branch length between nodes indicates genetic distance based on the nucleotide differences of four housekeeping genes of *V. alginolyticus*. The colors of nodes represent meat type. Dark blue nodes, seafood; light blue nodes, freshwater products; orange-yellow, others.

**Figure 7:**
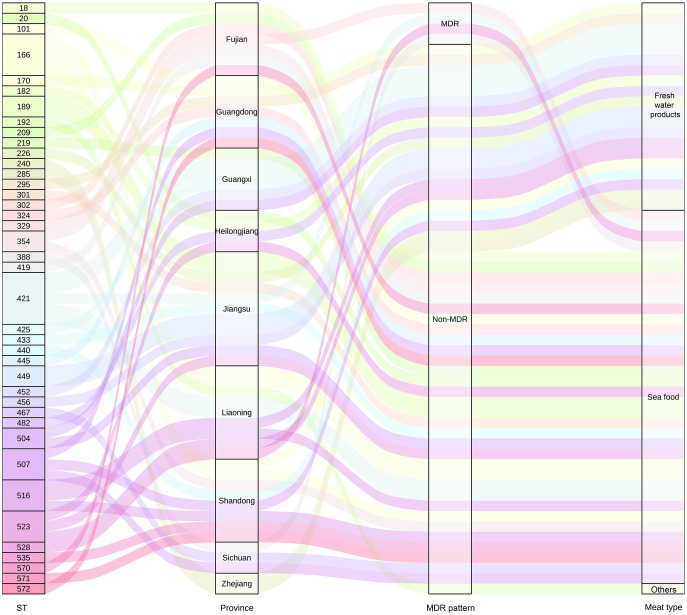
Distribution of *V. alginolyticus* isolates from retail meats is tracked using the Sankey plot. The number of *V. alginolyticus* isolates is indicated by the height of the rectangle. The line indicates the distribution of the STs in different provinces; drug resistance and meat types are colored by different STs.

Based on sequence typing (ST) prediction, 34 STs were detected in 55 *V. alginolyticus*. Overall, ST421 (*n* = 5), ST166 (*n* = 4), ST523 (*n* = 3), ST516 (*n* = 3), and ST507 (*n* = 3) were dominant STs among 57 *V. alginolyticus* isolates (Figure 6). It was observed that alignments of two *V. alginolyticus* isolates exhibited less than 90% similarity in allele length. Specifically, *V. alginolyticus* isolates from seafood (*n* = 36) showed 26 STs, among which ST421 (*n* = 3, 8.33%), ST166 (*n* = 3, 8.33%), and ST507 (*n* = 3, 8.33%) were the most common. In contrast, common STs of *V. alginolyticus* isolates from freshwater products (*n* = 19) were ST516 (*n* = 2, 10.53%), ST421 (*n* = 2, 10.53%), and ST328 (*n* = 2, 10.53%). The Sankey plot showed a widespread prevalence and high diversity of *V. alginolyticus* during the distribution analysis of each variable (Figure 7).

## 4 Discussion

*E. coli* and *Vibrio* species, which are the most common bacterial pathogens in the world, pose a serious risk to public health (Chan et al., 2003; Bresee et al., 2012). Fish proteins are widely used in pharmaceuticals, nutraceuticals, and food applications (Xu et al., 2020). China is currently the largest fish consumer, producer, and retailer of seafood products in the world (Villasante et al., 2013). Over the past three decades, marine fish products with high values have become increasingly appreciated in China (Chai and Bi, 2022). Moreover, aquatic products are a popular food in China due to their taste and nutritional value (Chen et al., 2018).

However, annually approximately 800 million meals of potentially contaminated filter-feeding shellfish/bivalves and other seafood products are raw or lightly steamed (Shuval, 2003). The transmission of pathogenic bacteria in fish marketed for human consumption has implications for the fish market and consumer health (Chai and Bi, 2022). The residues of antibiotics in farmed fish influence consumers’ perceptions of the wholesomeness and safety of farmed fish over wild fish (Claret et al., 2014). Therefore, it is crucial to monitor Vibrio contamination for the safety of fish-derived food.

The isolation of *V. alginolyticus* strains with multiple antibiotic resistance has been reported in several recent outbreaks (Mechri et al., 2015; Mohamad et al., 2019b). Multiple antibiotic resistance (MAR) strains of *V. alginolyticus* have caused severe economic setbacks to the aquaculture industry (Mohamad et al., 2019b). Our study revealed that *V. alginolyticus* had a higher level of antibiotic resistance, which contrasts with previous research findings. For example, some studies have reported similar detection for antibiotic sensitivity of *V. alginolyticus*. The detection of *V. alginolyticus* in Hong Kong was 49.02% (25/51), all strains were sensitive to ceftriaxone, and almost all were sensitive to ceftazidime, chloramphenicol, and sulfamethoxazole except one or two strains. Most isolates were resistant to ampicillin (60.8%, 31/51), cefuroxime (66.7%, 34/51), and kanamycin (58.8%, 30/51) (Li et al., 1999). More than 80% of *Vibrio* isolates were resistant to ampicillin, and 2.5% of *V. alginolyticus* were resistant to ceftazidime and cefotetan in Italy (Zanetti et al., 2001). On the west coast of Korea, the detection of *V. alginolyticus* was 17.78% (16/90) and all *V. alginolyticus* were resistant to ampicillin and sensitive to gentamicin, tetracycline, and chloramphenicol (Kang et al., 2016). Some *Vibrio* species develop and are no longer susceptible to ampicillin, cefotaxime, tetracycline, and chloramphenicol (Li et al., 1999; Zanetti et al., 2001). In our study, two isolates (2020RZ128 and 2020RZ167) (1.56%, 2/128) of *V. alginolyticus* were resistant to gentamicin, two isolates (2020RZ111 and 2020RZ129) (1.56%, 2/128) were resistant to tetracycline, and one isolate (2020RZ129) (7.8%, 1/128) was resistant to chloramphenicol.

The use of antimicrobials in aquaculture may cause the potential risk of resistance, its transfer into the aquatic environment, and the selection of resistant strains (Balebona et al., 1998). Multidrug resistance of *Vibrio* species posed a major challenge for health practitioners and a huge threat to human health. Among 128 *V. alginolyticus* isolates, 8 (6.25%) isolates were demonstrated multiple resistance. Isolates collected from freshwater products had a higher level of antibiotic resistance (7.8%, 4/51) than marine foods (4%, 3/75). Variation in the prevalence of *V. alginolyticus* was likely attributable to the sampling sites, water salinity, seasons of the year, species of fish, postharvest practices, and hygienic standards applied during the handling, transport, and storage of seafood products (Lee et al., 2008; Jun et al., 2012; Abd-Elghany and Sallam, 2013).

Antibiotic resistance mechanisms of *Vibrio* and other bacterial species included mutation, acquisition of resistance-conferring plasmids/episomes, modification or degradation of target sites, altered drug uptake/altered membrane permeability, and induction or upregulation of drug efflux (Blair et al., 2015; Acosta-Smith et al., 2017; Teschler et al., 2017; Bina et al., 2018; Rajpara et al., 2018). On the other hand, the deregulation of critical cellular metabolic pathways, including carbon and energy metabolism, plays a role in or can modulate the resistance of pathogenic bacteria to small-molecule drugs, including antibiotics (Peng et al., 2015; Gutierrez et al., 2017; Su et al., 2018). Furfural and malonate, inhibitors of pyruvate dehydrogenase and succinate dehydrogenase (P cycle enzymes), increased resistance of isogenic ceftazidime-resistant *V. alginolyticus* to antibiotics (Liu et al., 2019).

The prevalence of antibiotic resistance among *Vibrio* species highlights the adaptive mechanisms bacteria have developed to overcome antimicrobial agents. Studies from various geographical regions demonstrate a wide range of resistance patterns to commonly used antibiotics. For instance, high levels of resistance to ampicillin have been observed in *Vibrio* isolates, indicating a significant challenge in the treatment of infections caused by these pathogens. Moreover, the emergence of resistance to other antibiotics such as ceftazidime, cefotetan, and even tetracycline underscores the dynamic nature of bacterial resistance and its implications for therapeutic strategies. This variability in resistance patterns across different locations and *Vibrio* strains reflects the complex interplay between bacterial genetics, antibiotic usage, and environmental factors.

In our study, 13 antibiotic-resistant genes were detected. As the most commonly used drug in poultry, the resistance of tetracycline is mediated by more than 40 acquired tetracycline-resistant genes, which encode for either efflux pumps, enzymatic inactivation, or ribosomal protection genes (Møller et al., 2016). *Tet* gene encoded a tetracycline efflux (van den Bogaard and Stobberingh, 2000; Reynolds et al., 2020); 97.1% (99/102) of isolates carried the *tet* gene, while two isolates are resistant to tetracycline. Given the observed resistance to tetracycline among *Vibrio* species, understanding the molecular mechanisms behind this phenomenon becomes crucial. Proteins encoded by *tet* genes confer tetracycline resistance to bacteria via multiple mechanisms: active efflux of tetracycline (efflux pumps) (Zhu et al., 2018), modification of the tetracycline binding site to inhibit binding (Li et al., 2013), and enzymatic deactivation of tetracycline (Jahantigh et al., 2020). These strategies ensure bacterial survival in the presence of tetracycline-class antibiotics, representing a significant facet of bacterial adaptive evolution.

Urinary tract infections (UTIs) are among the most common bacterial infections (Ortega-Lozano et al., 2023). The antibiotic Bactrim, or trimethoprim-sulfamethoxazole, is commonly used in the treatment of UTIs (DeMasi et al., 2021). It operates by sulfamethoxazole inhibiting bacterial folate synthesis (Iftikhar et al., 2023), while trimethoprim blocks the utilization of folate by bacteria (Janssen et al., 2015), collectively halting bacterial proliferation. However, the increasing reports of resistance to Bactrim have raised significant concerns. In our findings, five strains were resistant to Bactrim, while two isolates carried the *sul* gene. The emergence of resistance diminishes the therapeutic efficacy of Bactrim, complicating the treatment of UTIs. This not only poses a direct threat to patient health but also exacerbates the burden on healthcare resources.

It has been reported that fluoroquinolone resistance can be acquired through mutations in the quinolone resistance gene *qnrS* as well as mutations in the quinolone resistance determining region (QRDR) containing the *gyrA*, *gyrB*, *parC*, and *parE* genes (Chiou et al., 2014; Ingle et al., 2019). In our study, the isolate that carried the *qnrS* gene was resistant to ciprofloxacin. In total, 96% of isolates that carried the *blaCARB* gene were resistant to ampicillin. Interestingly, the isolate (2020RZ111) which carried the *floR* gene was sensitive to chloramphenicol, the same as two isolates (2020RZ79 and 2020RZ111) that carried *aph(3″)-Ib* gene and *aph(6)-Id* gene were sensitive to aminoglycoside.

The antimicrobial susceptibility testing in this study revealed differences in resistance among strains from various sources, highlighting the risk posed by *Vibrio* to food safety and public health. Our analysis showed that resistance is primarily influenced by the presence and expression levels of specific genes. Whole-genome sequencing and MLST typing demonstrated genetic similarities among strains, providing insight into their spread across different regions and food sources. This study emphasizes the importance of investigating strain diversity and distribution in the context of global food production and supply chains.

*V. alginolyticus* has been the second most common *Vibrio* species since 2007 due to increasing overall vibriosis rates (CDC 2014). Due to the significant economic losses caused by *V. alginolyticus*, the identification of its virulence genes has attracted increasing attention. However, most of the studies conducted on *V. alginolyticus* in the last 20 years focused on virulence-based genes. Type III secretion system (T3SS) is a major virulence factor that delivers effectors into the host eukaryotic cytoplasm (Lian et al., 2021). Basically, T3SS are ring-like structures embedded within a basal membrane and hollow needles formed from polymerized proteins. *vscU*, *vscS*, *vscR*, and *vcrD* genes encoded the T3SS C-ring protein. *Vsco* encoded T3SS protein. *vscN* encoded T3SS ATPase. *vscI*, *vscF*, *tyeA*, and *vcrH* encoded T3SS inner rod protein, needle protein, regulatory protein, and chaperone, respectively. *vpoR* encoded T3SS effector. *vopD* and *vopB* encoded T3SS translocator protein. In our study, we found that 54.4% (31/57) of isolates carried *vopD*, *vopB*, *vcrH*, and *tyeA* simultaneously. Additionally, 50.9% (29/57) of isolates carried the *vscI* and *vscF* genes, simultaneously, while 40.4% (23/57) of isolates simultaneously carried *vscS*, *vscR*, and *vscN*. Thermolabile hemolysin (tlh) is the primary virulence determinant of Vibrio (Meza et al., 2022). In our study, 42.1% (24/57) of isolates carried *tlh* gene, although *tlh* gene is known as a species marker for *V. parahaemolyticus* (Mohamad et al., 2019a). Different species of *Vibrio* form cohesive groups within which they easily exchange genetic elements to confer greater antibiotic resistance as well as regulate virulence (Ashrafudoulla et al., 2019).

## 5 Conclusion

*Vibrio alginolyticus* has been identified as prevalent in both freshwater products and marine foods, with certain isolates displaying resistance to multiple antibiotics alongside carrying the T3SS-related gene and the virulence gene *tlh*. This multifaceted antibiotic resistance highlights freshwater products and marine foods as potential vectors for *V. alginolyticus* infections in humans. Currently, the management of bacterial infections within aquaculture heavily relies on antibiotics, raising concerns about environmental impact and consumer health. Given the paramount importance of food safety in public health, there is an urgent need for the rational application of antibiotics in the aquaculture industry and stringent control measures against *V. alginolyticus* in retail meat products.

Our research highlights the significant ramifications of antibiotic resistance in *V. alginolyticus* on both public health and aquaculture methodologies. It advocates for intensified monitoring, judicious application of antibiotics, and stringent adherence to food safety standards to diminish the hazards posed by antibiotic-resistant organisms. This investigation delineates a strategic approach for ensuring food safety and fortifying public health in China, accentuating the imperative for a collaborative endeavor to adeptly navigate antibiotic resistance within aquacultural environments.

## Data availability statement

The data has been uploaded to the bioproject PRJNA1090258 on https://www.ncbi.nlm.nih.gov/bioproject.

## Author contributions

YS: Data curation, Visualization, Formal analysis, Writing – original draft. YY: Data curation, Visualization, Formal analysis, Writing – original draft. SY: Data curation, Visualization, Formal analysis, Software, Writing – review & editing. FL: Resources, Investigation, Methogology, Writing – review & editing. YL: Resources, Investigation, Methogology, Writing – original draft. LY: Resources, Investigation, Methogology, Writing – original draft. DY: Resources, Investigation, Methogology, Writing – original draft. ZP: Resources, Investigation, Methogology, Writing – original draft. BY: Investigation, Methogology, Writing–original draft. JS: Investigation, Methogology, Writing – original draft. JX: Investigation, Methogology, Writing – original draft. YD: Investigation, Methogology, Writing – original draft. YB: Data curation, Conceptualization, Methodology, writing – review & editing, Funding acquisition.

## Footnotes

^1^ https://www.genomicepidemiology.org/
